# Novel Transcatheter Edge-to-Edge Repair System for High-Risk Primary Mitral Regurgitation: 2-Year Pivotal Trial Outcomes

**DOI:** 10.1016/j.jacasi.2026.04.029

**Published:** 2026-06-25

**Authors:** Yucheng Zhong, Ying Zhi, Changdong Zhang, Chao Cheng, Zhijiang Xie, Qingwei Ji, Yuan Bai, Xiaoping Peng, Gicheng Xi, Jiancheng Xiu, Fei Ma, Xiaoke Shang, Yan Wang, Nianguo Dong

**Affiliations:** a Department of Cardiovascular Surgery, Union Hospital, Tongji Medical College, Huazhong University of Science and Technology, Wuhan, China; b Department of Cardiology, People's Hospital of Guangxi Zhuang Autonomous Region, Nanning, China; c Department of Cardio-Metabolic Medicine, Renmin Hospital, Wuhan University, Wuhan, China; d Department of Cardiology, Xiangyang First People's Hospital, Hubei University of Medicine, Xiangyang, China; e Department of Cardiology, Handan First Hospital, Handan, China; f Department of Cardiology, Changhai Hospital Affiliated to Naval Medical University, Shanghai, China; g Department of Cardiology, The First Affiliated Hospital of Nanchang University, Jiangxi Hypertension Research Institute, Nanchang, China; h Department of Cardiac Surgery, Shanxi Cardiovascular Hospital, Taiyuan, China; i Department of Cardiovascular Medicine, Nanfang Hospital, Southern Medical University, Guangzhou, China; j Cardiovascular Clinical Medical Center, Liyuan Hospital, Tongji Medical College, Huazhong University of Science and Technology, Wuhan, China; k Department of Cardiology, Xiamen Cardiovascular Hospital, School of medicine, Xiamen University, Xiamen, China

**Keywords:** mitral valve clip, primary mitral regurgitation, prospective multicenter trial, transcatheter edge-to-edge repair

## Abstract

**Background:**

Transcatheter edge-to-edge repair (TEER) is established for severe primary mitral regurgitation (MR), but robust clinical data in Asian populations are limited.

**Objectives:**

This study aimed to evaluate the safety and efficacy of the novel Neonova TEER system in high-risk Chinese patients with symptomatic, moderate-to-severe or severe primary MR.

**Methods:**

This prospective, multicenter, single-arm, performance-goal pivotal trial enrolled 123 patients from 23 Chinese centers (April 2022 to March 2024). Eligible patients had symptomatic primary MR (≥3+) and high surgical risk. The primary efficacy endpoint was treatment success at 1 year (freedom from mortality, mitral valve surgery, and MR >2+) compared with a 60% performance goal. Follow-up extended to 2 years.

**Results:**

Event-free survival at 1 year was 92.68%, exceeding the target value, and remained 84.55% at 2 years. The proportion of patients with MR ≤2+ was 96.61% at 1 year and 95.41% at 2 years. Immediate procedural success was 98.37%. The proportion of patients in NYHA functional class I/II improved from 0% at baseline to 94.92% at 1 year, sustained at 92.52% at 2 years. Significant left ventricular reverse remodeling occurred. The composite major adverse event rate was 8.13% at 1 year, with all-cause mortality at 2.44%. All-cause mortality at 2-year follow-up was only 4.07%.

**Conclusions:**

The novel TEER system demonstrates excellent safety and efficacy for high-risk primary MR patients, achieving high event-free survival and sustained clinical and echocardiographic improvements through 2 years. It offers a reliable, minimally invasive alternative to conventional surgery.

Mitral regurgitation (MR), the most prevalent valvular heart disease worldwide, represents a growing health care burden due to an aging population. Significant MR affects 1.6% to 2.0% of the general population, rising sharply to 10% among individuals older than 75 years.^1^^,^^2^ In the United States, approximately 1.3 million adults currently have MR, with this number projected to increase to 2.3 million by 2030.^3^ Although comprehensive national data from China are lacking, single-center studies suggest a potentially higher prevalence, estimating 10 million patients with severe MR.^4^ The condition leads to progressive cardiac remodeling, eventually resulting in heart failure, substantially impaired quality of life, and increased mortality rates.^5^

Surgery is traditionally the standard treatment for MR, but many patients cannot tolerate surgical procedures because of advanced age, comorbidities, or high operative risk. This unmet clinical need led to the development of transcatheter edge-to-edge repair (TEER), a transformative minimally invasive procedure offering an effective alternative for patients unsuitable for strategy.^6^^,^^7^ The landmark EVEREST II (Endovascular Valve Edge‑to‑Edge Repair Study II) trial demonstrated the efficacy of TEER and highlighted the importance of patient selection.^8^ The EXPAND G4 study further improved the safety and efficiency of TEER, enhanced treatment standards, and broadened the eligible patient population.^9^ These studies provide evidence supporting the Class IIa guideline recommendation for TEER in patients with high-risk primary MR.^10^ However, data from Asian populations, who may have distinct clinical characteristics, remain limited. The large patient population in China with MR presents a substantial unmet need for minimally invasive therapies.^11^ This situation has driven independent research and development of TEER devices in China to overcome anatomical limitations and accessibility barriers through technological innovation.

This prospective, multicenter, single-arm, performance-goal pivotal trial was designed to evaluate the safety and efficacy of a novel TEER system (Neonova, Hangzhou Duanyou Medical Technology Co, Ltd) in high-risk Chinese patients with primary MR. The TEER system features a linear design and an automatic elastic mechanism intended to reduce the risk of leaflet tearing and chordal rupture. Conducted across 23 centers from April 2022 to March 2024, the study competitively enrolled 123 patients aged ≥18 years with symptomatic severe primary MR (grade ≥3+) who were at high surgical risk. The primary efficacy endpoint was the 1-year event-free survival rate, with follow-up extended to 2 years to assess long-term efficacy and safety (Central Illustration). Secondary endpoints included immediate procedural success rate, incidence of surgical intervention due to mitral valve (MV) dysfunction, proportion of patients with NYHA functional class I or II, and cardiovascular rehospitalization rate. Safety endpoints comprised the incidence of major adverse events (MAEs), all-cause mortality, and cardiovascular mortality. This trial provides multicenter registrational clinical evidence for the TEER system in China, expanding the global evidence base and addressing the specific needs of Asian populations.

**Central Illustration:**
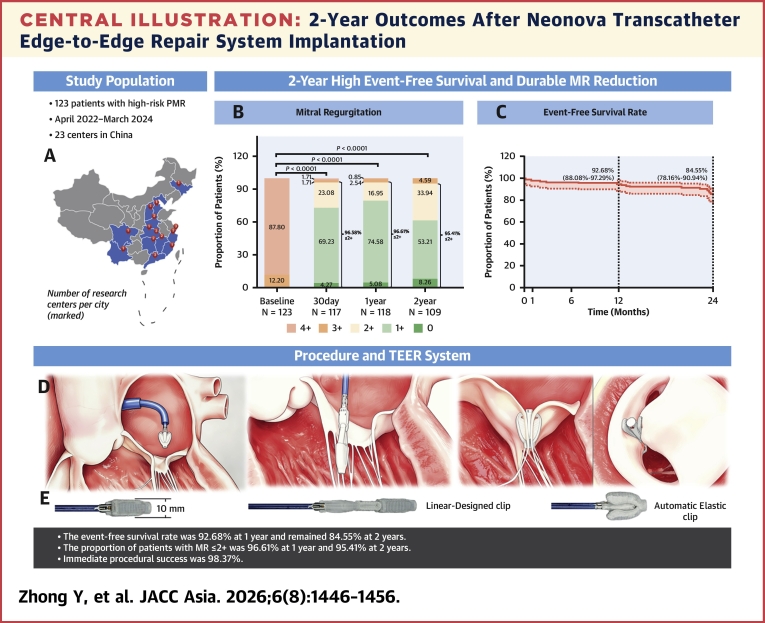
2-Year Outcomes After Neonova Transcatheter Edge-to-Edge Repair System Implantation (A) Geographic distribution of the 23 study centers in China. (B) Relative frequency of mitral regurgitation (MR) severity grades at baseline compared with 30 days (n = 117; MR ≤2+ = 96.58%), 1 year (n = 118; MR ≤2+ = 96.61%), and 2 years (n = 109; MR ≤2+ = 95.41%). Graphs present unpaired data. *P* values were calculated from paired analyses using the Wilcoxon signed-rank test, with *P* < 0.05 considered significant. (C) Kaplan-Meier analysis estimating the 2-year event-free survival rate, with corresponding 95% CIs. (D) Hand-drawn illustration of the transcatheter edge-to-edge repair (TEER) system implantation workflow. (E) Photograph of the clip, featuring 10-mm size, linear design, and automatic elastic mechanism. PMR = primary mitral regurgitation.

## Methods

### Study design and participants

This prospective, multicenter, single-arm, pivotal trial evaluated the safety and efficacy of the TEER System in patients with moderate-to-severe or severe primary MR. The single-arm performance-goal design was adopted based on extensive clinical data from similar marketed products, as the trial device has no essential differences.

Outcomes were reported for 123 patients enrolled from 23 centers in China, with follow-up assessments conducted predischarge and at 30 days, 6 months, and 1 year (primary efficacy endpoint). Follow-up continued through 2 years with evaluations including echocardiography, heart functional class, and MAE. The study protocol was designed by the principal investigators, steering committee, and sponsor (Hangzhou Duanyou Medical Technology Co, Ltd). The study protocol (Version 1.0, dated January 4, 2022) and informed consent forms were approved by the Ethics Committee of Union Hospital, Tongji Medical College, Huazhong University of Science and Technology (one of the lead sites) on April 7, 2022 (approval no. UHCT-IEC-SOP-016-03-01). All patients provided written informed consent, and local approvals were obtained at all participating centers. The study was registered with the Chinese Clinical Trial Registry (ChiCTR2200060182). Additional details are provided in Supplemental Table 1).

Surgical risk was assessed using the Society of Thoracic Surgeons (STS) Predicted Risk of Mortality calculator.^12^ Detailed definitions of comorbid conditions included in the STS risk score (diabetes, infective endocarditis, hypertension, chronic lung disease, peripheral vascular disease, cerebrovascular disease, coronary artery disease, and arrhythmia) are provided in the Supplemental Appendix.

### Inclusion and exclusion criteria

Eligible participants were adults (≥18 years) with symptomatic heart failure (NYHA functional class II–IV) and moderate-to-severe or severe primary MR (≥3+ by transthoracic echocardiography). All participants were deemed unsuitable for MV surgery by the local heart team due to elevated surgical risk (STS score ≥8 for replacement or ≥6 for repair, >2 frailty indices, or dysfunction of >2 organs). In addition, candidates needed appropriate mitral anatomy for edge-to-edge repair, feasible femoral venous access, and provided written informed consent.

Key exclusion criteria included untreated significant coronary artery disease or recent coronary intervention (within 30 days); concomitant severe aortic valve disease (≥3+) requiring surgery; unsuitable MV anatomy (eg, severe calcification, prior surgery, or rheumatic disease); presence of left heart thrombus or vegetation; severe left ventricular dysfunction (left ventricular ejection fraction [LVEF] <20%) or dilation (left ventricular end-diastolic diameter [LVEDD] >7.0 cm); prohibitive venous access; contraindications to antithrombotic therapy or major bleeding within 3 months; active infection or endocarditis; other comorbidities limiting follow-up or outcome assessment; pregnancy or breastfeeding; concurrent participation in another clinical trial; or investigator-assessed poor compliance.

### Procedures

The novel TEER system comprises an implantable clip (model specifications: TMrCLAMP, TMrCLAMP-M, TMrCLAMP-S), an adjustable steerable guide sheath, and a delivery catheter system. Procedures were performed via femoral venous access with transseptal puncture. Under transesophageal echocardiographic and fluoroscopic guidance, the clip was positioned across the MV, deployed to grasp the leaflets, and released to create a double-orifice, reducing regurgitation (Central Illustration). Multiple clips could be implanted if necessary.

Postprocedure antithrombotic management was individualized. Patients without prior indications for anticoagulation received dual antiplatelet therapy for 1 to 6 months, followed by single-agent maintenance. Patients requiring anticoagulation (eg, atrial fibrillation) continued their oral regimen, with antiplatelet therapy added based on bleeding risk. All regimens were assessed at follow-up.^13^^,^^14^

### Evaluation criteria

The primary composite endpoint event comprised all-cause mortality, surgical intervention for MV dysfunction, or postprocedural MR grade >2+. The primary effectiveness endpoint was the proportion of patients free from this composite endpoint at 1 year. Secondary effectiveness endpoints included the rate of immediate procedural success (defined as successful device implantation with postprocedural MR ≤2+ and no in-hospital death or surgical reoperation), the incidence of subsequent surgical intervention for MV dysfunction, the proportion of patients achieving NYHA functional class I or II, and the rate of cardiovascular rehospitalization, with the latter 3 assessed at 30 days, 6 months, and 1 year post procedure.

Safety endpoints, assessed at discharge, 30 days, 6 months, and 1 year, included incidence of MAE (a composite of death, stroke, myocardial infarction, renal failure, or cardiovascular surgery related to the device or procedure after transseptal puncture), all-cause mortality, and cardiovascular mortality.

Extended follow-up was conducted through 2 years post procedure to evaluate long-term device durability and clinical status. Assessments during this phase focused on echocardiographic evaluation of MV function, tracking the occurrence of the primary composite endpoint events, and serial assessment of NYHA functional class.

### Statistical analysis

Descriptive statistics are presented as counts (proportions) for categorical variables and mean ± SD or median (IQR) for continuous variables, as appropriate. Comparisons between 2 time points were performed using paired Student’s *t*-tests or Wilcoxon signed-rank tests. The primary efficacy endpoint was analyzed in the full analysis set using the Kaplan-Meier method, with missing data imputed by the worst-rank method. The 95% CI was calculated using the normal approximation, and its lower limit was compared against a prespecified performance goal. Secondary efficacy and safety endpoints were analyzed using appropriate descriptive and inferential statistical methods based on data type. Adverse events were summarized by frequency and incidence. A 2-sided alpha level of 0.05 was used to determine statistical significance. All analyses were conducted using SAS version 9.4. Additional details regarding statistical methods are provided in the Supplemental Materials.

## Results

### Baseline characteristics of subjects

**Figure 1:**
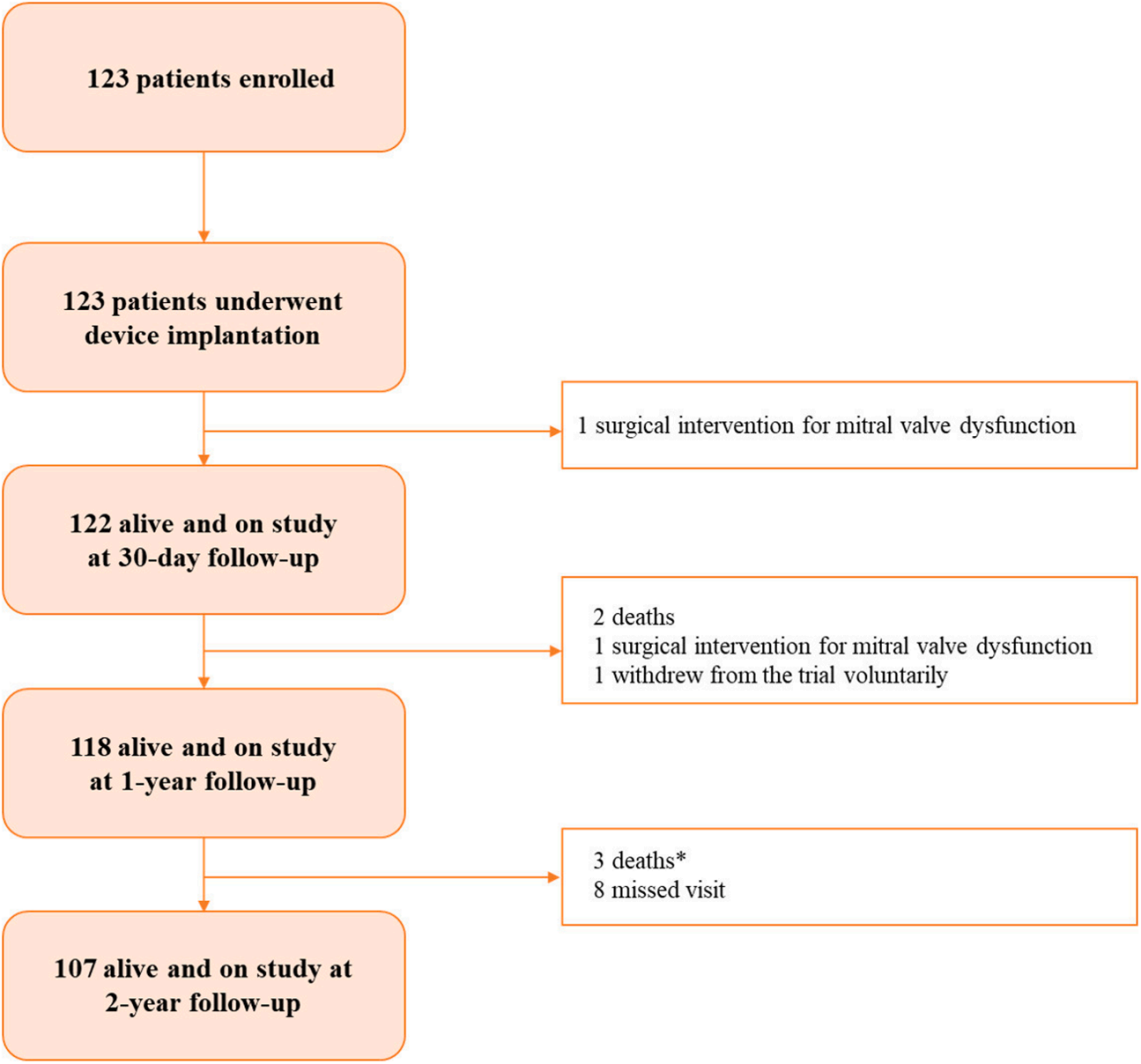
Patient Disposition and Flow Subject enrollment, follow-up visits, and events at 30 days (window, −7 to +7 days), 1 year (window, −45 to +45 days), and 2 years (window, −45 to +45 days) are shown. ∗One patient completed the 1-year visit but died within the 1-year window; 2 patients died after the 1-year window but before the 2-year follow-up.

From April 26, 2022, to March 21, 2024, a total of 123 patients were enrolled across 23 centers in China (Figure 1, Central Illustration, Supplemental Table 2). A total of 118 patients successfully completed the 1-year follow-up, and 1 patient voluntarily withdrew from the trial. Follow-up data were available for 109 patients (88.62 %) at 2 years (Figure 1).

The 123 enrolled subjects had a mean age of 70.79 ± 6.75 years, with 67 men (54.47%) and 56 women (45.53%). The average height and weight were 160.86 ± 8.59 cm and 60.20 ± 12.88 kg, respectively. Comorbidities included hypertension (61.79%), chronic pulmonary disease (53.66%), coronary artery disease (52.85%), peripheral vascular disease (52.85%), atrial fibrillation (41.46%), cerebrovascular disease (37.40%), renal insufficiency (31.71%), diabetes mellitus (15.45%), and previous cardiac surgery (2.44%). Surgical risk analysis showed that the mean STS score for MV repair (%) was 7.72% ± 1.54% (n = 7), and the mean STS score for MV replacement was 7.75% ± 3.79% (n = 116). Baseline characteristics are shown in Table 1.

**Table 1:** Baseline Characteristics (N = 123)

Age, y	70.79 (6.75)
Sex	
Male	67 (54.47%)
Female	56 (45.53%)
Height, cm	160.86 ± 8.59
Weight, kg	60.20 ± 12.88
STS score, %	
For mitral valve repair (n = 7)	7.72 ± 1.54
For mitral valve replacement (n = 116)	7.75 ± 3.79
NYHA functional class	
I	0 (0.00%)
II	20 (16.26%)
III	87 (70.73%)
IV	16 (13.01%)
Comorbidities	
Hypertension	76 (61.79%)
Chronic pulmonary disease	66 (53.66%)
Coronary artery disease	65 (52.85%)
Previous myocardial infarction	4 (3.25%)
Previous percutaneous coronary intervention	11 (8.94%)
Previous coronary artery bypass grafting	2 (1.63%)
Peripheral vascular disease	65 (52.85%)
Atrial fibrillation	51 (41.46%)
Cerebrovascular disease	46 (37.40%)
CKD stage ≥III^a^	39 (31.71%)
Diabetes mellitus	19 (15.45%)
Previous cardiac surgery	3 (2.44%)
Rheumatic heart disease	0 (0.00%)
Left ventricular structure and function	
Left ventricular ejection fraction, %	62.68 ± 8.14
Left ventricular end-diastolic diameter, mm (n = 121)	54.32 ± 6.74
Left ventricular end-diastolic volume, mL (n = 113)	142.26 ± 49.05
Left ventricular end-systolic diameter, mm (n = 121)	35.97 ± 6.12
Left ventricular end-systolic volume, mL (n = 113)	54.49 ± 24.25
Mitral regurgitation severity	
Moderate-to-severe, grade 3+	15 (12.20%)
Severe, grade 4+	108 (87.80%)
Effective regurgitant orifice area (n = 117)	0.57 ± 0.22
Left atrial diameter, mm (n = 121)	47.66 ± 7.99
Mean transmitral valve pressure gradient (n = 117)	2.20 ± 1.11
Vena contracta width, A-P, mm (n = 121)	8.99 ± 2.37
Tricuspid regurgitation grade ≥3+	22 (17.88%)
Lesion location	
Central	74 (60.16%)
Noncentral	49 (39.84%)
Pulmonary artery systolic pressure, mm Hg (n = 117)	43.18 ± 17.74
Clinically significant abnormal proBNP / BNP /NT-proBNP^b^	59 (47.97%)
proBNP, pg/ml (n = 8)	659.8 (126.5-895.3)
BNP, pg/ml (n = 37)	95.0 (29.7-312.0)
NT-proBNP, pg/mL (n = 75)	433.7 (153.5-1697)

Values are n (%), mean ± SD, and median (Q1-Q3).

A-P = anterior-posterior; FAS = full analysis set; STS = Society of Thoracic Surgeons; BNP = B-type natriuretic peptide; proBNP = prohormone of BNP; NT-proBNP = N-terminal pro–B-type natriuretic peptide. Clinically significant abnormal BNP/proBNP/NT-proBNP.

a Chronic kidney disease (CKD) stage ≥III, defined as estimated glomerular filtration rate <60 mL/min/1.73 m^2^.

b Clinically significant abnormality of BNP/proBNP/NT-proBNP is defined by the physician, integrating quantitative test results with the patient’s clinical presentation, age, renal function, and other relevant individual factors.

All patients were assessed as high-risk surgical candidates with moderate-to-severe or severe primary MR by the heart team. Preoperatively, 87 patients (70.73%) were classified as NYHA functional class III, and 16 patients (13.01%) as class IV. Among them, 59 patients (47.97%) had clinically significant abnormal preoperative natriuretic peptide (B-type natriuretic peptide [BNP]/pro–B-type natriuretic peptide [proBNP]/ N-terminal pro–B-type natriuretic peptide [NT-proBNP]) levels. Echocardiographic evaluation showed a mean LVEF of 62.47% ± 8.68%, LVEDD of 54.31 ± 6.74 mm, and left ventricular end-diastolic volume of 142.26 ± 49.05 mL. Left ventricular end-systolic diameter and end-systolic volume were 35.97 ± 6.12 mm and 54.49 ± 24.25 mL, respectively. MR severity was grade 3+ in 15 patients (12.20%) and grade 4+ in 108 patients (87.80%). The mean effective regurgitant orifice area was 0.57±0.22 cm^2^, mean left atrial diameter was 48.3 ± 7.2 mm, and mean mitral transvalvular pressure gradient 2.20 ± 1.11 mm Hg (Table 1, Supplemental Table 3). Of the 123 patients, 22 (17.88%) had more than moderate tricuspid regurgitation and mean pulmonary artery systolic pressure was 43.18 ± 17.74 mm Hg (Table 1). Lesion locations were central (A2/P2) in 89 patients (72.4%) and noncentral (commissural or multisegment) in 34 patients (27.6%). The most common site of MV prolapse was the P2 region, present in 59 patients (47.97%). In addition, 65 patients (53.28%) had flail MV (Supplemental Table 3).

### Procedural details

A total of 174 clips were implanted in 123 subjects, including 86 TMrCLAMP clips and 88 TMrCLAMP-S clips. Among them, 75 patients (60.98%) required implantation of a single clip to achieve satisfactory repair outcomes. For more complex cases, 45 patients (36.58%) required 2 clips, and in highly complex scenarios, 3 patients (2.44%) required 3 clips (Table 2). Notably, the device implantation success rate was 100%, with no intraoperative conversions to surgery or periprocedural deaths (Table 2). One periprocedural complication occurred: an anterior leaflet clip dislodged during the second clip implantation. This was successfully resolved by implanting a third clip without adverse patient outcomes. At 1-year follow-up, MR in this patient improved to grade 2+ (Table 2, Supplemental Table 4). Intraoperative echocardiography was used to assess immediate post‑procedural mitral regurgitation parameters, which served as the primary indicator for determining procedural success (Table 3).

**Table 2:** Procedural Details (N = 123)^a^

Successful implantation	123 (100.00%)
Number of devices implanted	
1	75 (60.98%)
2	45 (36.58%)
3	3 (2.44%)
Primary device model	
TMrCLAMP	77 (62.60%)
TMrCLAMP-M	0 (0.00%)
TMrCLAMP-S	46 (37.40%)
Second/third device model	
TMrCLAMP	9 (17.65%)
TMrCLAMP-M	0 (0.00%)
TMrCLAMP-S	42 (82.35%)
Procedure time, skin incision to closure, min	140.00 (84.00-190.00)
Device deployment time, min^b^	58.00 (31.00-121.00)
Intraoperative conversion to surgery	0 (0.00%)
Periprocedural complication	1 (0.81%)

Values are n (%), or median (Q1-Q3).

Abbreviation as in Table 1.

a Transseptal puncture (TSP) height was not systematically recorded in the original case report forms. Based on operator experience and published anatomical/guideline consensus, the recommended TSP height ranges (measured from the mitral annular plane) for successful device placement according to the target scallop region are as follows: A1/P1 (anterior): approximately 3.8–4.1 cm; A2/P2 (central): approximately 4.0–4.3 cm; A3/P3 (posterior): approximately 4.4–4.7 cm. Individual patient data are not available.

b Device implantation time was defined as the time from the insertion of the clip delivery system into the sheath to its final release and detachment.

### Efficacy assessment

The primary effectiveness endpoint was the 1-year event-free survival rate, defined as the proportion of patients free from the composite primary endpoint (all-cause mortality, surgery for valve dysfunction, or MR grade >2) at 1 year. The 1-year Kaplan-Meier estimate for event-free survival rate was 92.68% (95% CI: 88.08%-97.29%), exceeding the target value of 60% (Table 4, Central Illustration C). Nine patients failed to meet the primary effectiveness endpoint: 3 died (1 after completing the 1-year follow-up within the visit window), 2 underwent MV surgery due to valve dysfunction (surgical sternotomy to remove the clip and implant a bioprosthetic valve), 1 withdrew voluntarily (data imputed via the worst-case scenario), and 3 patients had MR grade >2+ at 1 year (2 with grade 3+and 1 with grade 4+). The extended follow-up to 2 years provided additional insights into the long-term efficacy of the mitral clip implantation. At 2 years, event-free survival rate remained stable at 84.55% (95% CI: 78.16%-90.94%) (Central Illustration C).

**Table 3:** Intraoperative Echocardiography (N = 123)

Mitral regurgitation severity	
No, grade 0	6 (4.88%)
Trace, grade 1+	93 (75.61%)
Moderate, grade 2+	22 (17.89%)
Moderate-to-severe, grade 3+	2 (1.63%)
Severe, grade 4+	0 (0.00%)
Effective regurgitant orifice area (n = 85)	0.10 ± 0.08
Vena contracta width, A-P, mm (n = 122)	2.25 ± 1.05
Stability of the clip	123 (100.00%)

Values are n (%) or mean ± SD.

Abbreviations as in Table 1.

**Table 4:** Clinical Outcome

	Before Discharge	30 Days	1 Year	2 Years^a^
Primary efficacy endpoint	--	--	92.68% (88.08%-97.29%)	84.55% (78.16%-90.94%)
Secondary efficacy endpoints				
Immediate procedural success	121 (98.37%)	--	--	--
Mitral valve dysfunction-related surgeries^b^	1 (1.63%)	1 (1.63%)	2 (1.63%)	2 (1.63%)
NYHA functional class I or II	57 (46.34%)	100 (81.97%)	112 (94.92%)	--
Cardiovascular rehospitalization^c^		3 (2.44%)	14 (11.38%)	--
Safety evaluation indicators				
MAE^d^	1 (0.81%)	2 (1.63%)	10 (8.13%)	--
All-cause mortality	0 (0.00%)	0 (0.00%)	3 (2.44%)	5 (4.07%)
Cardiovascular mortality	0 (0.00%)	0 (0.00%)	1 (0.81%)	1 (0.81%)

Values are n (%) or % (95% CI).

Abbreviations as in Table 1.

a Extended follow-up included outcomes assessed at 2 years.

b Cumulative incidence analysis was performed.

c Cardiovascular rehospitalization encompassed readmission events attributable to various cardiovascular conditions, including heart failure, myocardial infarction, infective endocarditis, and third-degree atrioventricular block.

d Major adverse events (MAE) are defined as a composite endpoint of death, stroke, myocardial infarction, renal failure, and cardiovascular surgery due to device- or procedure-related adverse events occurring after transfemoral venous transseptal access establishment. 95% 2-sided CI was assessed using the normal approximation method.

Comparing baseline and follow-up echocardiographic findings, MR was significantly decreased and was maintained for at least 2 years (Central Illustration B). At 30 days, 96.58% of patients achieved MR grade ≤2+. At 1 year, this proportion was 96.61%, with 94 patients achieving MR ≤1+. At 2 years, 95.41% still maintained MR grade ≤2+, confirming sustained procedure efficacy. Secondary efficacy endpoints showed an immediate procedural success rate of 98.37% (Table 4). Intraoperative echocardiography revealed that following the implantation of the investigational device, MR was completely resolved in 6 patients (4.88%), reduced to grade 1+ in 93 patients (75.61%), and diminished to grade 2+ in 22 patients (17.89%) (Table 3). The effective regurgitant orifice area of the MV was 0.10 ± 0.08 cm^2^, and vena contracta width was 2.25 ± 1.05 mm. Stability of the clip was confirmed in all patients (Table 3).

**Figure 2:**
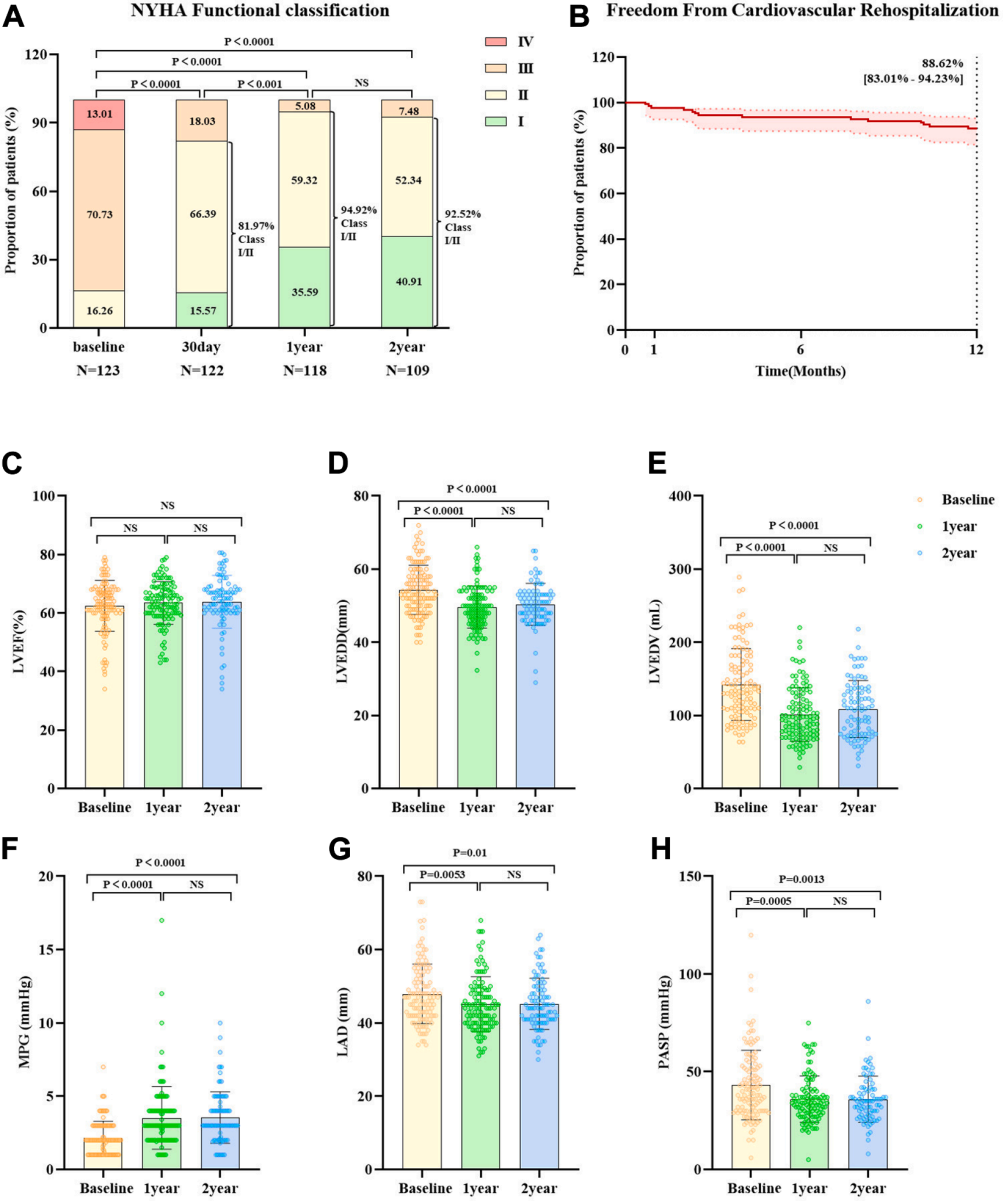
Changes in Cardiac Function and Remodeling (A) Relative frequency of NYHA functional classification at baseline compared with 30 days (n = 122), 1 year (n = 118), and 2 years (n = 109). Unpaired data shown. *P* values were calculated from paired analyses using the Wilcoxon signed-rank test, with *P* < 0.05 considered significant. (B) Kaplan-Meier analysis estimating the 1-year freedom from cardiovascular rehospitalization rate, with 95% CIs. (C) Left ventricular ejection fraction (LVEF), (D) left ventricular end-diastolic diameter (LVEDD), (E) left ventricular end-diastolic volume (LVEDV), (F) mitral valve transvalvular pressure gradient (MPG), (G) left atrial diameter (LAD), and (H) pulmonary artery systolic pressure (PASP) were assessed by echocardiography at baseline and 1-year and 2-year follow-up. Between-group comparisons were performed using Student’s *t*-test; *P* < 0.05 was considered significant.

Patients’ NYHA functional class demonstrated significant, sustained improvement from baseline following device implantation (Figure 2A, Table 4). The proportion achieving NYHA functional class I or II was 81.97% (100 of 122) at 30 days post procedure, rose significantly to 94.92% (112 of 118) at 1 year, and remained at 92.52% (101 of 109) at the 2-year follow-up. Paired analysis revealed functional class improvement from baseline in 104 patients (84.55%, 95% CI: 77.31%-90.26%) at 1 year and 97 patients (78.86%, 95% CI: 70.79%-85.61%) at 2 years. However, differences between the 1- and 2-year assessments were not significant (Figure 2A).

Cardiac systolic function and ventricular remodeling were further assessed by echocardiography at 1 and 2 years after device implantation (Figures 2C to 2H). No significant change in LVEF was observed from baseline to either follow-up time point. In contrast, significant reductions in LVEDD and left ventricular end-diastolic volume were observed at 1 year compared with baseline. These reductions were maintained at 2 years, with no significant difference between the 2 follow-up periods. As illustrated in Figures 2F-2H, MV transvalvular pressure gradient increased after device implantation but remained stable between 1 and 2 years. Left atrial diameter decreased significantly from baseline at both the 1-year and 2-year follow-ups, without significant change between these 2 assessments. Similarly, pulmonary artery systolic pressure showed a sustained reduction at 1 and 2 years compared with baseline, with no significant difference between the 2 follow-up evaluations. Collectively, these long-term follow-up results indicated reverse left ventricular remodeling, accompanied by favorable reductions in left atrial size and pulmonary pressure, following device implantation.

**Figure 3:**
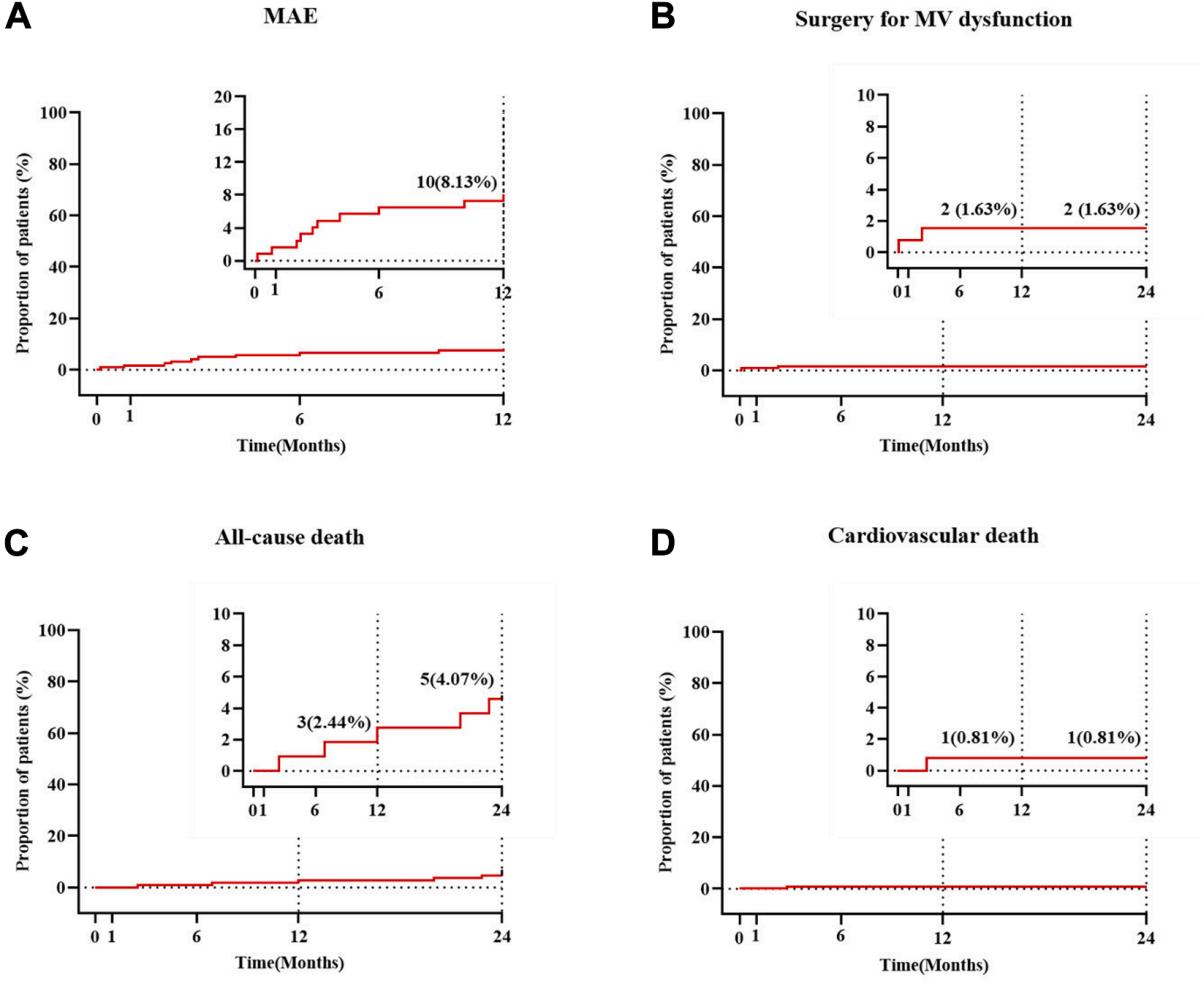
Kaplan-Meier Curves for Safety Endpoints Through Follow-up Kaplan-Meier analysis estimating the proportion of patients experiencing (A) a major adverse event (MAE), (B) surgery for mitral valve (MV) dysfunction, (C) all-cause death, and (D) cardiovascular death. Data are presented as numbers and percentages at specified postoperative time points (1 year for MAE; 1 and 2 years for other endpoints).

Surgery for MV dysfunction was extremely rare, required in only 2 patients (1.63%), one before discharge and another within 6 months post procedure (Figure 3B, Table 4). The 1-year cardiovascular readmission rate was 11.38% (95% CI: 5.70-16.90); corresponding data for the 2-year extended follow-up have not yet been obtained (Figure 2B, Table 4).

### Safety assessment

The safety profile of this trial was carefully evaluated based on the incidence of MAE, all-cause mortality, and cardiovascular mortality. MAE was defined as a composite endpoint including death, stroke, myocardial infarction, renal failure, and cardiovascular surgery due to device- or procedure-related adverse events occurring after the transseptal puncture via the femoral vein. The incidence of MAE was 0.81% (1 of 123) before discharge, 1.63% (2 of 123) at 30 days, 6.50% (8 of 123) at 6 months, and 8.13% (10 of 123) at 1 year postoperatively (Figure 3A, Table 4). Importantly, no deaths were reported within the first 30 days post procedure. At the 1-year follow-up, the all-cause mortality rate was 2.44% (3 of 123), with a cardiovascular mortality rate of 0.81% (1 of 123). At the 2-year follow-up, the all-cause mortality rate was 4.07% (5 of 123), without additional cardiovascular deaths (Figures 3C and 3D, Table 4).

## Discussion

This study enrolled 123 patients from 23 centers and provides strong evidence for the safety and efficacy of the TEER system in treating patients with moderate-to-severe or severe primary MR. The event-free survival rate at 1 year postoperatively was 92.68%, substantially exceeding the prespecified target value of 60%, which underscores the clinical effectiveness of this intervention (Central Illustration A and C). This result aligns with reported rates for comparable international devices (eg, MitraClip: 93.8%)^15^^,^^16^ and surpasses that of a major domestic device (Dragonfly: 87.5%),^17^ underscoring the clinical effectiveness of this intervention. This success rate is comparable to or even exceeds traditional surgical interventions, particularly in high-risk patients who are often considered unsuitable for open-heart surgery due to comorbidities or advanced age.^18^

Compared with established TEER devices, the novel TEER system utilizes a “linear-designed” and “automatic elastic” clip design that prevents chordae entanglement and clamps leaflets firmly while protecting them from damage. The system features a reduced delivery profile, minimal device intrusion depth (approximately 2-3 mm), and a maximum 10-mm clip size, reducing the need for multiple clips (Central Illustration C and D) In Asian patients, who typically have smaller left atrial dimensions,^19^ these design features may facilitate transseptal crossing, reduce the risk of chordal entanglement, and improve maneuverability. Although head-to-head comparative studies are lacking, the current findings suggest potential advantages of the novel TEER system for Asian patients with complex or small cardiac anatomy.

Our findings are consistent with previous studies on percutaneous interventions for MR, such as those employing the MitraClip system. The immediate procedural success rate of 98.37% observed in our study matches the high success rates reported in other trials, highlighting the reliability of transcatheter interventions.^20^ The low incidence of MAE and favorable safety profile further support the clinical utility of this technology.^21^ The 1-year all-cause mortality rate of 2.44% and cardiovascular mortality rate of 0.81%are particularly noteworthy, indicating minimal postoperative complications and high patient survival rates.^7^

Extended follow-up at 2 years showed a stable success rate of 83.73%, confirming procedural durability. This long-term efficacy is crucial for patients with primary MR, who often face chronic health challenges and require sustained improvements in their quality of life.^22^ Sustained stability in the proportion of patients with MR ≤2+ beyond 1 year, confirmed by echocardiography, further demonstrates the long-term functional durability of the clip. The significant improvement in NYHA functional class sustained over 2 years indicates substantial enhancement in patient quality of life.^23^

Detailed procedural data highlight the versatility and adaptability of the TEER system. The capability to deploy multiple clips, as necessary, to achieve optimal valve function underscores the flexibility of this approach.^24^ The 100% device implantation success rate, without intraoperative conversion to surgery, further validates the procedural reliability. The single periprocedural complication, which was successfully resolved without adverse effects, demonstrates the robustness of the system and the effectiveness of the backup strategies.

### Study limitations

First, this single-arm study utilized a performance-goal design without a randomized comparator. The modest sample size was restricted to high-risk primary MR patients, and certain long-term outcomes, including cardiovascular rehospitalization beyond 1 year, were not fully captured. Second, transseptal puncture (TSP) height was not prospectively collected as a prespecified variable in the case report forms. Despite post hoc efforts to retrieve these data from the participating centers, individual patient measurements could not be obtained. Thus, quantitative TSP height values or related correlative analyses with procedural outcomes are unavailable. However, based on operator experience and published consensus, recommended TSP height ranges according to the target scallop region have been provided in Table 2 as a general reference. In addition, patient quality of life was not assessed.

### Perspectives

Future investigations should include larger cohorts with extended follow-up to comprehensively evaluate long-term efficacy, complication risks, and quality-of-life impacts. Comparative studies with conventional surgical approaches and alternative percutaneous interventions are needed to establish optimal treatment strategies for diverse patient populations. Future studies should systematically record TSP height to clarify its impact on TEER success and safety.

## Conclusions

The novel TEER system provides a safe, effective, and durable treatment for patients with primary MR. The high success rates, low complication rates, and significant improvements in quality of life indicate that this intervention is a promising alternative to traditional surgical methods, particularly for high-risk patients. As transcatheter interventions continue to evolve, this study establishes a strong foundation for further advancements in treating MR.

## Funding Support and Author Disclosures

This work was supported by the Hubei Provincial Science and Technology Program Project (Grant No. 2025BCB114). The data supporting the findings of this study are available from the sponsor but restrictions apply to their availability. Data are, however, available from the authors on reasonable request and with the permission of the sponsor, for the purpose of collaborative research that has received prior approval through a research proposal. The authors have reported that they have no relationships relevant to the contents of this paper to disclose.
