# Supplementary material for: Novel Transcatheter Edge-to-Edge Repair System for High-Risk Primary Mitral Regurgitation: 2-Year Pivotal Trial Outcomes
Source: JACC Asia. 2026 Jun 25;6(8):1446–56. doi: 10.1016/j.jacasi.2026.04.029 (PMC13458933; doi:10.1016/j.jacasi.2026.04.029)
Supplement: Supplementary file 1 [file mmc1.docx]

**Supplementary Materials**

**Methods**

**1. Trial Method Selection and Rationale**

This clinical trial was conducted according to NMPA regulations, including the Administrative Measures for Medical Device Registration and Good Clinical Practice for Medical Device Clinical Trials. A single-arm performance goal design was selected based on the following rationale:

Extensive clinical trial data exist for similar approved devices. MitraClip® has completed 18 registration trials (e.g., EVEREST I, EVEREST II, EVEREST II RCT, EVEREST II High Risk, EVEREST II Realism, COAPT, TRAMI, ACCESS-EU) involving populations in Europe, America, and the Asia-Pacific region. These trials provided robust evidence-based support for MitraClip® and analogous devices. MitraClip® has been approved by the FDA and NMPA for mitral regurgitation. The FDA approved its use for high-risk degenerative MR in 2013 and functional MR in 2019. As of October 2019, more than 80,000 MitraClip® procedures had been performed globally, confirming its safety and effectiveness. The investigational Neonova™ system is substantially equivalent to the approved MitraClip® device. Therefore, a single-arm performance goal design was considered appropriate.

**2. Statistical Analysis Methods**

2.1. Statistical Design, Methods, and Analysis Procedures

2.1.1. Statistical Analysis Sets

Full Analysis Set (FAS): Defined according to the intention-to-treat principle, comprising all enrolled subjects who received the investigational product.

Safety Set (SS): Defined as all subjects enrolled in the trial who received the investigational product and had at least one safety assessment.

The primary efficacy analysis, baseline demographics, and all secondary efficacy analyses will be performed using the FAS. Safety evaluations will be conducted using the SS.

2.1.2. Research Hypothesis

This study employs a prospective, multicenter, single-arm performance goal design. Comparison against a performance goal will demonstrate whether the investigational device meets clinical requirements. The primary efficacy endpoint is the 1-year treatment success rate (defined as freedom from all-cause death, mitral valve surgery due to valvular dysfunction, and MR ≤ 2+ at 1-year follow-up). The corresponding statistical hypothesis is as follows:

𝛨_0_: 𝑃_𝑇_ ≤ 𝑃_0_

𝛨_1_: 𝑃_𝑇_ > 𝑃_0_

Where 𝑃_𝑇_ represents the 1-year treatment success rate of the investigational product, and 𝑃_0_ represents the performance goal.

2.1.3. Statistical Analysis

1) Descriptive Analysis: Categorical data are presented as frequencies and percentages. Continuous data are summarized using the mean, standard deviation, median, 25th and 75th percentiles, as well as minimum and maximum values.

2) Analysis of Baseline Demographics: The number of subjects enrolled and those who completed the study at each center are summarized. Discontinued cases are listed, detailing their distribution across centers, discontinuation rates, and reasons for discontinuation. Patient demographics (e.g., age, height, vital signs), medical history, and medication history are described.

3) Efficacy Analysis: In addition to descriptive methods, the primary efficacy endpoint is analyzed using the Kaplan-Meier method to estimate event rates and associated 95% confidence intervals (with patients lost to follow-up treated as censored). The lower limit of the confidence interval is compared with a pre-specified performance goal to determine if the study device meets clinical application requirements.

Secondary efficacy endpoints are analyzed using appropriate statistical methods based on data characteristics.

4) Safety Evaluation: Adverse events are summarized according to the number of occurrences and incidence rates. All adverse events are listed in detail, including their clinical presentations, severity, and relationship to device implantation.

5) Statistical Considerations: Analysis of the primary endpoint is conducted at a one-sided 0.025 significance level (corresponding to a one-sided 97.5% confidence limit). Analyses of secondary endpoints are generally performed at a two-sided 0.05 significance level, unless otherwise specified. All statistical analyses are conducted using SAS® software, version 9.4.

2.1.4. Statistical Analysis Procedures

All statistical analyses are conducted in compliance with the ICH E9 guidelines and the Guiding Principles for Biostatistics in Clinical Trials issued by the National Medical Products Administration (NMPA). Additionally, all statistical procedures strictly follow Standard Operating Procedures (SOPs) established by the Peking University Clinical Research Institute.

2.2. Performance Goal and Sample Size

The planned enrollment includes 120 patients. This sample size was determined according to statistical principles, considering the study hypothesis and projected efficacy based on the primary endpoint. Due to limited clinical data for similar products, international studies of the MitraClip® in corresponding patient populations served as primary references. These included the EVEREST I study, reporting an efficacy endpoint of 66% (J Am Coll Cardiol 2009;54:686-94); the EVEREST II High Risk Study with 59.3% (J Am Coll Cardiol 2012;59:130-9); the EVEREST II study with 55% (N Engl J Med 2011;364:1395-406); and the Prohibitive Risk DM analysis (page 27) from the MitraClip® US FDA premarket approval submission reporting 61.4%. The reported 1-year all-cause mortality rate after MitraClip® treatment across these trials ranged from 6.1% to 17.3%. For conventional mitral valve surgery, the corresponding rate ranged from 0.9% to 21%, and the incidence of mitral regurgitation grade ≥3+ ranged from 18.3% to 21.1%.

Based on aggregated data from comparable populations, and assuming the investigational device’s efficacy is similar to published MitraClip® results in patients deemed prohibitive surgical risk, the 1-year treatment success performance goal was set at 60%, consistent with the MitraClip® literature.

Pilot study results projected a 1-year treatment success rate of 73%. With a performance goal of 60%, one-sided α = 0.025, 80% power, and a maximum attrition of 10%, the calculated sample size was 120 patients. The sample size formula is as follows:

$$n=\frac{{[Z_{1-\alpha/2}\sqrt{P_{0}(1 - P_{0})} + Z_{1-\beta}\sqrt{P_{T}(1 - P_{T})}]}^{2}}{{(P_{T}-P_{0})}^{2}}$$

where 𝑃_𝑇_ represents the projected 1-year treatment success rate, 𝑃_0_ represents the performance goal, α represents the Type I error rate (set at 0.025), and β represents the Type II error rate (set at 0.2, corresponding to 80% power).

2.3. Clinical Trial Result Pass/Fail Criteria

The trial’s success or failure is determined statistically by verifying the initial hypothesis. In this study, the primary endpoint is the 1-year treatment success rate. The clinical utility of the investigational product is demonstrated by comparing the observed results against a predefined performance goal.

Trial outcomes are evaluated by comparing the observed 1-year treatment success rate with the performance goal. If the lower bound of the 95% confidence interval for the 1-year treatment success rate exceeds the predefined goal of 60%, the result is considered positive, confirming that the product meets clinical requirements.

2.4. Missing Data Handling

In the FAS, missing data for the primary efficacy endpoint are imputed using the worst-case imputation method.

3. **Ethical Statement**

This clinical trial strictly adhered to ethical principles governing medical research involving human subjects, as outlined in the Declaration of Helsinki, Good Clinical Practice (GCP), and relevant national regulatory requirements. The Institutional Review Board of Union Hospital, Tongji Medical College, Huazhong University of Science and Technology, as one of the lead sites, approved the study protocol (Version 1.0 dated January 4, 2022) and informed consent forms on April 7, 2022. All other participating sites obtained ethical approval before initiating the trial.

During the trial, one protocol amendment was introduced. The Institutional Review Board of Xiamen Cardiovascular Hospital of Xiamen University, as the coordinating lead site, approved Protocol Version 2.0 (dated June 6, 2022) on August 16, 2022, before other centers. Subsequently, all other participating sites accepted or approved the amended protocol. All screened and enrolled subjects provided signed informed consent.

**4. Definitions of Comorbidities in STS Risk Score**

These definitions are aligned with the STS Adult Cardiac Surgery Database specifications.

**Diabetes:** Patients were defined as having diabetes if they had a history of diabetes (Type I or II) diagnosed or treated by a healthcare provider. Patients were categorized according to the most aggressive glycemic control therapy used at home: (a) **no diabetes**; (b) **diet-controlled** (dietary management alone); (c) **oral medication** (use of oral hypoglycemic agents); (d) **insulin-dependent** (use of insulin, with or without oral agents); (e) **other SubQ** (use of non-insulin subcutaneous injectable agents, such as GLP-1 receptor agonists); (f) **other control** (any other specified glucose-lowering methods not included in the above categories); or (g) **no control** (no active glucose-lowering therapy despite a known diabetes diagnosis).

**Infective Endocarditis:** Infective endocarditis was defined as a history of endocarditis diagnosed and documented by a healthcare provider, including the type of endocarditis. The disease was considered active if the patient was currently receiving antibiotic treatment (other than prophylactic medication) for endocarditis, regardless of blood culture positivity. This definition also included patients with preoperative endocarditis scheduled to begin antibiotics after surgery. If no antibiotic treatment (other than prophylactic medication) was administered at the time of surgery and cultures were negative, the infection was considered treated.

**Hypertension:** Patients were defined as hypertensive if they had a history of hypertension diagnosed and treated with medication, diet, or exercise, or if they were currently receiving pharmacological treatment for hypertension.

**Chronic Lung Disease (CLD):** Patients were defined as having CLD if they had documented CLD graded by severity as mild (FEV1 60-75%, DLCO 61% to lower limit of normal, or chronic inhaled/oral bronchodilator therapy), moderate (FEV1 50-59%, DLCO 40-60%, or chronic oral/systemic steroid therapy), or severe (FEV1 <50%, DLCO <40%, or room air pO_2_ <60 mmHg or pCO_2_ >50 mmHg). Patients with documented CLD but unspecified severity were categorized as “CLD present, unknown severity,” and those with missing information as “unknown.” Patients without these findings were classified as having no CLD.

**Cerebrovascular disease (CVD):** Patients were classified as having CVD if they had any of the following conditions, listed by priority: (a) stroke (CVA) within 30 days before surgery; (b) CVA occurring more than 30 days before surgery (remote stroke); (c) prior CVA with unknown timing (uncertain if ≤30 or >30 days); (d) transient ischemic attack (TIA); or (e) any other CVD not included in (a) to (d). Category (e) includes ≥50% stenosis of major extracranial or intracranial vessels supplying the brain (e.g., vertebral artery disease, internal carotid artery disease, or intracranial artery disease), previous cervical or cerebral artery revascularization surgery or percutaneous intervention, cerebral aneurysm, or occlusion of the vertebral, internal carotid, or intracranial artery due to dissection. Subdural hematoma or arteriovenous malformation (AVM) were not considered CVD. Chronic nonvascular neurological diseases or acute neurological insults (e.g., metabolic or anoxic ischemic encephalopathy) were also excluded.

**Peripheral vascular disease (PVD):** Patients were considered to have PVD if they had a documented history of peripheral arterial disease involving the upper or lower extremities, renal, mesenteric, or abdominal aortic systems. This includes: (a) claudication (either exertional or at rest); (b) amputation for arterial vascular insufficiency; (c) vascular reconstruction, bypass surgery, or percutaneous intervention involving the extremities (excluding dialysis fistulas and vein stripping); (d) documented abdominal aortic aneurysm (with or without repair); (e) documented subclavian artery stenosis; (f) documented chronic stable abdominal aortic dissection; or (g) documented peripheral artery aneurysms. PVD did not include diseases of the carotid arteries, cerebrovascular arteries, or thoracic aorta. It also excluded deep vein thrombosis (DVT), pulmonary artery aneurysm, Raynaud's disease, and AVM.

**Coronary artery disease (CAD):** Patients were defined as having CAD if they had a documented history of myocardial infarction (MI), percutaneous coronary intervention (PCI), coronary artery bypass grafting (CABG), or coronary angiography demonstrating ≥50% stenosis in any major epicardial vessel. For elective patients, the most severe coronary symptom experienced between admission and entry to the operating room (the presenting symptom leading to hospitalization) was recorded. The timing of the most recent MI relative to surgery (≤6 hours, >6–24 hours, 1–21 days, or >21 days) and the number of diseased major native coronary vessels (once diseased, always considered diseased) were documented.

**Arrhythmia:** Patients were classified as having an arrhythmia if they had a preoperatively diagnosed cardiac rhythm disorder, including atrial fibrillation, atrial flutter, ventricular tachycardia/ventricular fibrillation, sick sinus syndrome, second-degree atrioventricular block, or third-degree atrioventricular block. Arrhythmias were further classified as **remote** (occurring >30 days before surgery) or **recent** (occurring within 30 days before surgery) based on the timing of the most recent episode.

**Table S1. Specific Ethical Approval Details**

| Site Number | Site Name | Protocol V1.0 Ethics Approval Date | Protocol V2.0 Ethics Approval Date |
| --- | --- | --- | --- |
| 01 | Union Hospital, Tongji Medical College, Huazhong University of Science and Technology | 2022/4/26 | 2022/9/4 |
| 02 | Xiamen Cardiovascular Hospital, Xiamen University | 2022/5/7 | 2022/8/16 |
| 03 | Fuwai Yunnan Cardiovascular Hospital | 2022/7/8 | 2022/11/25 |
| 07 | Guangdong Provincial People's Hospital | 2022/7/22 | 2022/10/20 |
| 09 | Second Xiangya Hospital, Central South University | 2022/6/28 | 2022/10/28 |
| 10 | The First Affiliated Hospital of Naval Medical University (Changhai Hospital) | 2022/06/20 | 2022/9/30 |
| 13 | The First Affiliated Hospital of Zhengzhou University | 2022/7/4 | 2023/2/8 |
| 15 | Shanxi Cardiovascular Hospital | 2022/6/13 | 2022/10/27 |
| 16 | Tongji Hospital, Tongji Medical College, Huazhong University of Science and Technology | 2022/6/23 | 2022/12/28 |
| 17 | The First Affiliated Hospital of Nanchang University | 2022/8/11 | 2022/11/2 |
| 19 | Henan Provincial Chest Hospital | 2022/6/28 | 2022/12/22 |
| 21 | The First Affiliated Hospital, Zhejiang University School of Medicine | 2022/9/8 | 2022/12/8 |
| 23 | Shanghai Tenth People’s Hospital | 2022/7/13 | 2022/10/28 |
| 29 | Jiangxi Provincial People's Hospital | 2022/8/1 | 2022/10/24 |
| 31 | The Third Xiangya Hospital of Central South University | 2022/6/23 | 2022/10/20 |
| 32 | Handan First Hospital | 2022/6/12 | 2023/1/14 |
| 33 | Zhongnan Hospital of Wuhan University | 2022/7/15 | 2022/11/11 |
| 35 | People's Hospital of Sichuan Province | 2022/9/22 | 2022/10/17 |
| 36 | The First Affiliated Hospital, Sun Yat-sen University | 2022/9/16 | 2022/11/8 |
| 37 | Nanfang Hospital, Southern Medical University | 2022/9/19 | 2022/10/13 |
| 38 | Xiangyang First People's Hospital | 2022/7/29 | 2022/10/21 |
| 40 | The First Hospital of Jilin University | 2022/8/18 | 2022/11/25 |
| 43 | Putuo District Central Hospital Affiliated to Tongji University | NA | 2022/12/9 |

**Table S2 Distribution of Enrolled Subjects by Center**

| Site Number | Site Name | FAS | SS | Dropout Number | Dropout Rate |
| --- | --- | --- | --- | --- | --- |
| 01 | Union Hospital, Tongji Medical College, Huazhong University of Science and Technology | 32 | 32 | 2 | 6.25% |
| 02 | Xiamen Cardiovascular Hospital, Xiamen University | 20 | 20 | 0 | 0.00% |
| 03 | Fuwai Yunnan Cardiovascular Hospital | 9 | 9 | 0 | 0.00% |
| 07 | Guangdong Provincial People's Hospital | 5 | 5 | 0 | 0.00% |
| 09 | Second Xiangya Hospital, Central South University | 2 | 2 | 0 | 0.00% |
| 10 | The First Affiliated Hospital of Naval Medical University (Changhai Hospital) | 2 | 2 | 0 | 0.00% |
| 13 | The First Affiliated Hospital of Zhengzhou University | 1 | 1 | 0 | 0.00% |
| 15 | Shanxi Cardiovascular Hospital | 3 | 3 | 0 | 0.00% |
| 16 | Tongji Hospital, Tongji Medical College, Huazhong University of Science and Technology | 6 | 6 | 1 | 16.67% |
| 17 | The First Affiliated Hospital of Nanchang University | 4 | 4 | 0 | 0.00% |
| 19 | Henan Provincial Chest Hospital | 8 | 8 | 0 | 0.00% |
| 21 | The First Affiliated Hospital, Zhejiang University School of Medicine | 8 | 8 | 0 | 0.00% |
| 23 | Shanghai Tenth People’s Hospital | 1 | 1 | 0 | 0.00% |
| 29 | Jiangxi Provincial People's Hospital | 3 | 3 | 0 | 0.00% |
| 31 | The Third Xiangya Hospital of Central South University | 1 | 1 | 0 | 0.00% |
| 32 | Handan First Hospital | 5 | 5 | 0 | 0.00% |
| 33 | Zhongnan Hospital of Wuhan University | 2 | 2 | 0 | 0.00% |
| 35 | People's Hospital of Sichuan Province | 1 | 1 | 0 | 0.00% |
| 36 | The First Affiliated Hospital, Sun Yat-sen University | 2 | 2 | 0 | 0.00% |
| 37 | Nanfang Hospital, Southern Medical University | 2 | 2 | 0 | 0.00% |
| 38 | Xiangyang First People's Hospital | 1 | 1 | 0 | 0.00% |
| 40 | The First Hospital of Jilin University | 3 | 3 | 0 | 0.00% |
| 43 | Putuo District Central Hospital Affiliated to Tongji University | 2 | 2 | 0 | 0.00% |
| Total | | 123 | 123 | 3 | 2.44% |
| *1.The number of dropouts refers to subjects who did not complete the 1-year postoperative follow-up. Death is a primary efficacy endpoint, and deaths occurring within the 1-year postoperative follow-up period are not considered dropouts.*  *2.Subjects 01022 and 07005 were withdrawn from the trial due to death within the 1-year postoperative follow-up period and were considered to have completed the 1-year postoperative follow-up. Subject 02001 died after completing the 1-year postoperative follow-up, and the time of death (408 days postoperatively) was within the follow-up window (410 days postoperatively).*  *3.FAS = full analysis set, SS = safety set* | | | | | |

**Table S3 Transthoracic Echocardiography Findings at Screening**

| Program | | FAS |
| --- | --- | --- |
| Mitral valve prolapse location-A1 | | 12/123(9.76%) |
| Mitral valve prolapse location-A2 | | 24/123(19.51%) |
| Mitral valve prolapse location-A3 | | 13/123(10.57%) |
| Mitral valve prolapse location-P1 | | 30/123(24.93%) |
| Mitral valve prolapse location-P2 | | 59/123(47.97%) |
| Mitral valve prolapse location-P3 | | 47/123(38.21%) |
| Mitral valve leaflet calcification and cleft | | 8/123(6.56%) |
| Flail mitral valve | | 65/122(53.28%) |
| Mitral valve prolapse width, mm | | 11.86(3.72)/120(3) |
| Mitral valve prolapse height, mm | | 6.25(2.19) / 118(5) |
| Mitral valve effective orifice area, cm^2^ | | 5.48(1.02)/116(7) |
| Mitral transvalvular pressure gradient, mmHg | | 2.20(1.11) /117(6) |
| Tricuspid regurgitation grade | |  |
|  | 0 | 8/123 (6.50%) |
|  | 1+ | 64/123 (52.03%) |
|  | 2+ | 29/123 (23.58%) |
|  | 3+ | 14/123 (11.38%) |
|  | 4+ | 7/123 (5.69%) |
|  | 5+ | 1/123 (0.81%) |
| Aortic regurgitation grade | |  |
|  | 0 | 51/123 (41.46%) |
|  | 1+ | 60/123 (48.78%) |
|  | 2+ | 12/123 (9.76%) |
|  | 3+ | 0/123 (0.00%) |
|  | 4+ | 0/123 (0.00%) |
| *Values are n/N (%) and mean (SD)/N(Missing). A=anterior; P=posterior; FAS=full analysis set.* | | |

**Table 4 Description of Periprocedural Complication Adverse Events**

| Adverse Event Name | Specific Details | Management | Outcome |
| --- | --- | --- | --- |
| Clip Detachment (Anterior Leaflet) | During implantation of the second mitral valve clip (TMrCLAMP-S) at the 6-12 o’clock position, the clip detached from the anterior leaflet upon deployment. The patient remained asymptomatic, with no immediate signs of hemodynamic compromise. | A third mitral valve clip (TMrCLAMP) was promptly implanted to address residual regurgitation. | No patient discomfort or complications were observed. Post-procedural transesophageal echocardiography confirmed successful implantation of the third clip, with mitral regurgitation reduced to grade 2+, appropriate clip positioning, and stable morphology. |
